# Application of Turbiscan Stability Index for the Preparation of Alumina Photocatalytic Membranes for Dye Removal

**DOI:** 10.3390/membranes13040400

**Published:** 2023-03-31

**Authors:** Marida Blasi, Catia Algieri, Sudip Chakraborty, Vincenza Calabrò

**Affiliations:** 1 Department of DIMES, University of Calabria, Via Pietro Bucci, Cubo 42A, 87036 Rende, Italy; 2 Institute on Membrane Technology, National Research Council of Italy (ITM–CNR), Via Pietro Bucci, Cubo 17C, 87036 Rende, Italy

**Keywords:** Turbiscan Stability Index, alumina tubular membrane, titanium dioxide, methyl orange

## Abstract

In this work, for the first time, the stability of the TiO_2_ suspensions used for the photocatalytic membrane preparation was studied by considering the Turbiscan Stability Index (TSI). The use of a stable suspension during the membrane preparation (by the dip-coating method) permitted a better dispersion of the TiO_2_ nanoparticles into the membrane structure due to a reduction of agglomerates formation. The dip-coating was performed on the macroporous structure (external surface) of the Al_2_O_3_ membrane to avoid large reduction of the permeability. In addition, the reduction of the suspension infiltration along the membrane’s cross-section allowed us to preserve the separative layer of the modified membrane. The water flux was reduced by about 11% after the dip-coating. The photocatalytic performance of the prepared membranes was evaluated using the methyl orange as a model pollutant. The reusability of the photocatalytic membranes was also demonstrated.

## 1. Introduction

Today, the discharge of wastewater, produced by textile, paper and food industries [1], causes serious health problems for humans and aquatic life, mainly due to the presence of synthetic dye in the effluents [2]. These chemical compounds are divided into different categories: acid, basic, direct, azoic, reactive and disperse dyes [3]. The azo dyes are widely used in the textile industry [4] and are very resistant. The traditional technologies used for their treatment are ineffective due to their elevated chemical stability [5]. Advanced oxidation processes (AOPs) represent an alternative route for the effective removal of pollutants present in the wastewater [6]. These processes produce highly oxidative radicals that are capable of degrading, in a non-selective way, organic pollutants, and converting them to mineralized species [7]. They can be divided into different classes: chemical (ozonation, peroxidation, Fenton), physical (sonolysis, cavitation), photochemical, photocatalytic (UV/solar/semi-conductors, UV/H_2_O_2_, UV/O_3_, etc.), and electrochemical processes (electrooxidation, electro-Fenton, etc.) [8]. The photo-processes favor the excitation of the electrons in a semiconductor by using UV and UV-Vis light [9]. In particular, the absorption of a photon, with energy equal or greater than the band gap, permits the electron’s excitation from the valance band to conduction band, leaving a positive hole (h^+^) in the valance band [9]. The electrons can reduce the pollutants or react with electron acceptors (as, for example, with O_2_ present in the water), reducing it to a superoxide radical anion (O_2_^−^). The surface of the photocatalyst at the valence band is positively charged and takes electrons from water to create hydroxyl radicals (OH˙) [10]. The radicals formed can degrade the pollutants present in the wastewater. Today, the photocatalyst deeply used is titanium oxide (TiO_2_), being non-toxic, inexpensive and having high efficiency [11]. This process exhibits some limitations, with the formation of agglomerates during the reaction with a decrease of the catalytic activity (reduction of the active sites) and a reduction of the incident light [12]. In addition, the recovery of the TiO_2_ nanoparticles (NPs) from the reaction medium makes the process expensive [13]. These drawbacks can be overcome with a photocatalytic membrane reactor (PMR) that combines the photocatalytic technology with membrane processes. A PMR can be designed in two main configurations: with suspended catalysts or ones immobilized in/on the membrane structure [14]. In the first configuration, the photocatalytic particles degrade the pollutants, and the membrane exhibits activities of confining the catalyst, the pollutant, and the intermediate species formed during the process [15]. This configuration ensures good contact between the catalytic sites and reactants, presenting higher efficiency than the immobilized one. However, the light penetration is reduced when the catalyst concentration is too high [15]. In addition, considering that the suspension is an unstable system, the NPs’ precipitation on the membrane surface causes a decline in the permeate flux and a decrease in the catalytic activity [16]. The PMR with the catalyst immobilized on/in the membrane permits an easy recovery of the catalyst and so its reuse. With this configuration, the fouling is mitigated, due to the self-cleaning property of the membrane surface [16]. However, with an immobilized catalyst, a reduction of the catalytic activity is detected, due to a reduction of the surface area [16]. Zhang et al. [17] studied the degradation of the reactive brilliant red by means of a suspended photocatalytic membrane reactor. The rejection of dye markedly depends on the deposition of TiO_2_ aggregate on the membrane surface. A comparison between the two different configurations of the PMR for the degradation of two azo dyes was achieved by Molinari and coworkers [18]. The experimental data evidenced that the PMR configuration with suspended photocatalytic nanoparticles was significantly more efficient than the configuration with the photocatalyst entrapped in the membrane.

Polymeric membranes are used in various applications, such as in gas separation processes, as well as water purification, desalination, and dialysis, being cheap and simply manufactured and processed [18]. Different papers have been published concerning wastewater treatment, and the effect of catalyst and pollutant concentrations, pH, light intensity, and membrane pore size on the catalytic activity has been deeply investigated [19,20,21,22,23,24]. Ceramic membranes seem more appropriate for wastewater treatment than polymeric ones, owing to their higher hydrophilicity that implies lower fouling and higher fluxes at low trans-membrane pressures [25], even if their application at the industrial scale is limited by high capital costs [26]. Deepracha et al. [27] demonstrated the possibility of removing phenol (21%) from water with a TiO_2_-Al_2_O_3_ membrane. A recent study used a TiO_2_–alumina membrane prepared with sol-gel spin coating method [28]. The dye degradation after 120 min was found to be equal to 36% [28].

In this study, high-flux ceramic photocatalytic membranes having a tubular configuration were prepared by using the dip-coating method. For the first time, the Turbiscan Stability Index was used for evaluating the stability of the suspensions employed for the photocatalytic membrane preparation. The photocatalytic activity of the prepared membranes was tested in a PMR for the degradation of methyl orange (MO), a recalcitrant azo dye. The reusability of the photocatalytic membranes during four successive reactions was also evaluated. Finally, the kinetics of MO degradation have been evaluated.

## 2. Materials and Methods

### 2.1. Materials

Titanium dioxide (TiO_2_; Honeywell Fluka, Seelze, Germany) was used as a photocatalyst. The additives used for the slurry stabilization were nitric acid (HNO_3_, Honeywell Fluka, pure) and polyvinylpyrrolidone (PVP, Carl Erba reagents, average molecular weight 17,000 Da). A tubular α-Al_2_O_3_ membrane (pore size: 200 nm, internal diameter.: 7 mm, outer diameter.: 10 mm; length: 10 cm; IKTS, Hermsdorf (Thuringia), Germany) was utilized for the preparation of the photocatalytic membranes. Methyl orange (Titolchimica S.p.a., Pontecchio Polesine (Ro) ITALY) was employed as a recalcitrant model dye. Distilled water was used to prepare the suspensions and for the membrane washing.

### 2.2. Suspension Preparation and Characterization

The morphology and size of the commercial TiO_2_ nanoparticles were observed by scanning electron microscope (SEM) using a Cambridge Zeiss LEO 400 microscope. The TiO_2_ powder was analyzed by X-ray diffractometry, using a Rigaku MiniFlex 600 X-ray diffractometer (Rigaku Corporation, Tokyo, Japan) with CuKα (wavelength of 1.5406 Å) radiation generated at 20 mA and 40 KV. The samples were scanned at 0.02 2θ at a rate of 1°/min, between 5°and 60° (2θ angle range).

To prepare the suspensions, an appropriate amount of TiO_2_ nanoparticles was dispersed in 30 mL of distilled water and stirred magnetically for 30 min at 30 °C (see Table 1). Subsequently, the effect of sonication and some additives on suspension stability was evaluated. In particular, the suspension after the magnetic stir (for 30 min) was sonicated for 20 min at 30 °C.

To obtain a suspension containing the PVP, used as adsorbate, 5 mL of the polymeric solution (two concentrations were considered: 0.2 wt. % and 0.8 wt. % [29]) was mixed with 25 mL of the TiO_2_ suspension (see Table 1), and stirred for 30 min at 30 °C. Finally, the effect of the nitric acid was evaluated by adding some drops of nitric acid solution (pH: 2.0) to the suspension containing titanium dioxide nanoparticles.

The suspension stability was studied by Turbiscan LAB^®^ (Formulation SAS, Versailles, France) at room temperature for 5 h, and the coating was carried out in the same time interval.

This instrument works by the use of light-scattering to detect particle migration and the formation of clusters in the liquid dispersion. It exhibits two detectors, working in transmission (*T*) and backscattering (BS) mode (*λ* = 880 nm), and, therefore, transparent and opaque samples were analyzed [30]. The equation used for calculating *T* is the following [7].

(1) $${T\left(\lambda ,r_{i}\right)=T_{0}e}^{-\frac{2r_{i}}{\lambda }}$$

and λ is given by:

(2) $$\lambda (d,\phi )=\frac{2d}{3\phi Q_{s}}$$

where *T* is the transmittance, *λ* the mean free-path of the photon, *r_i_* is the inner radius of the sample vial, *d* is the average size of the particle, *Φ* is the volume concentration of the dispersion phase, and *Q_s_* is an optical parameter. The backscattering is influenced by the size of the particles and volume concentration as reported in Equation (3).

(3) $$BS=\sqrt{\frac{3\phi (1-g)Q_{s}}{2d}}$$

Turbiscan LAB^®^ calculates the TSI, a parameter directly related to transmission and backscattering signals. The instrument calculates the TSI value by comparing each scan to the previous one at a selected height, and dividing the result by the total height (see Equation (4)) [31].

(4) $$T\mathrm{SI}=\sum_{i}\frac{\sum_{h}\left|{\mathrm{scan}}_{i}\left(h\right)-{\mathrm{scan}}_{i-1}\left(h\right)\right|}{H}$$

where scan_i_ (h) and scan_i-1_(h) are the values of the profile for a given scan “i” and the previous one “i-1” obtained at a given height “h”. H is the total height of the sample. The TSI values change in the range 0–100. The increase of TSI value indicates a loss of suspension stability.

Some suspensions’ zeta potential was measured using a Malvern Mastersizer 2000, Malvern Instruments. Before the analysis, particles were dispersed with an ultrasonication bath for 5 min.

### 2.3. Photocatalytic Membranes: Preparation and Characterization

**Figure 1:**
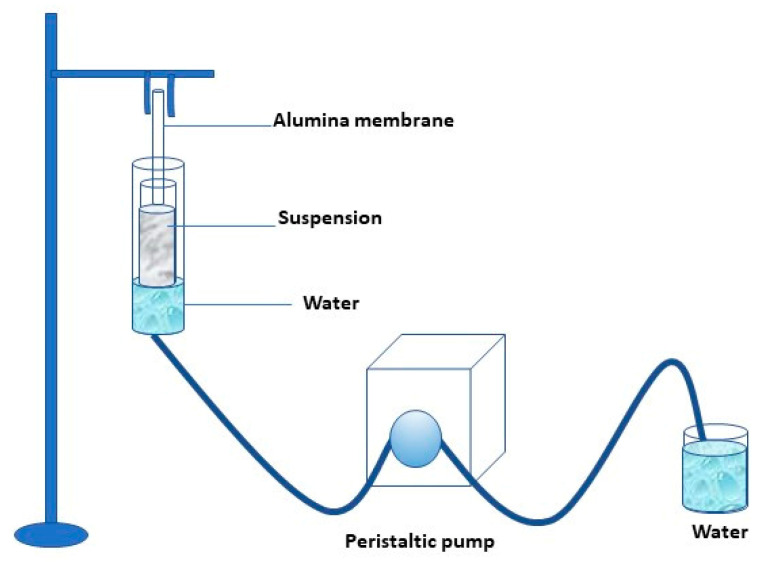
Scheme of the lab plant used for the preparation of the photocatalytic membrane.

Photocatalytic membranes were prepared by dipping the alumina membrane into the more stable TiO_2_ suspension. The coating occurred on the external surface of the ceramic membrane. In addition, the photocatalytic nanoparticles’ presence in the support’s internal surface was avoided by sealing the ends of the alumina tubular membranes with Teflon tape. The laboratory scale plant is schematized in Figure 1.

The membranes were dip-coated for 5 h, and the removal of the water from the glass tube was realized with the aid of a peristaltic pump at a feed flow rate of 0.2 mLs^−1^.The morphology of both pristine and photocatalytic membranes (indicated as TiO_2_-Al_2_O_3_ membrane) was observed by scanning electron microscope. Elemental analysis of the prepared membranes has been performed using energy dispersive X-ray (EDX) using a ZEISS crossbeam 350 instrument. The water flux through pristine and coated membranes has been measured under different trans-membrane pressures (0.2–1.6 bar), and the permeate flux was calculated by using Equation (5):

(5) $$J=\frac{V}{A\times t}$$

where *J* is the permeate flux (Lm^−2^ h^−1^), *V* is the volume of the accumulated permeate, *A* the membrane area, and *t* is the filtration time [32]. The hydraulic permeance was determined from the slope of water flux versus transmembrane pressure difference. Each experiment was carried out at least in triplicate.

### 2.4. Photocatalytic Membrane Tests

**Figure 2:**
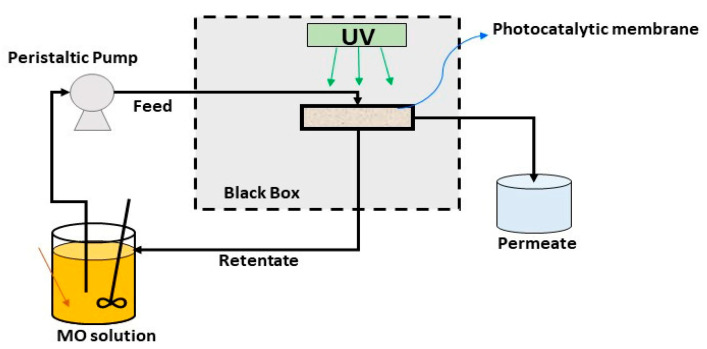
Scheme of the PMR used for MO degradation.

The laboratory scale plant used for assessing he photocatalytic activity of the TiO_2_-Al_2_O_3_ membranes is reported in Figure 2.

The TiO_2_–Al_2_O_3_ membranes (area = 17.59 cm^2^) were housed in a tubular glass membrane module (as shown in Figure 2), and placed in a light exposure chamber. The distance between the UV light and the membrane module was about 12 cm. The feed was 1 L of an aqueous solution of methyl orange (concentration = 1 mgL^−1^) and was recirculated through the system by a peristaltic pump. The PMR, operated in crossflow filtration mode, and the methyl orange degradation was performed continuously. In particular, the retentate has been collected in the feed tank, and the permeate in a separate container. The dye solution was exposed to ultraviolet (UV) light (λ = 365 nm) for 4 h.

The stability and reusability of the photocatalytic membranes were tested by reusing the same membranes (four reactions). The MO concentration was determined by measuring the absorbance at a wavelength of 464 nm, by using a UV-visible Lambda EZ201 spectrophotometer (PerkinElmer).

The degradation percentage of MO was calculated by using the Equation (6):

(6) $$\%Degradation=\frac{C_{0}-C_{t}}{C_{0}}\times 100$$

where *C*_0_ is the in initial concentration and *C_t_* the residual concentration after time *t*.

The kinetics of dye degradation was assessed considering the zero-order, first-order, and second-order models, and the Equations are reported below.

Zero-order kinetics:

(7) $$C_{t}-C_{0}=k_{0}t$$

First order kinetics:

(8) $$\ln\left(\frac{C_{0}}{C_{t}}\right)=k_{1}t$$

Second order kinetics:

(9) $$\frac{1}{C_{t}}-\frac{1}{C_{0}}=K_{2}t$$

where *k*_0_, *k*_1_, and *k*_2_ are the kinetic rate constants for zero-order, first-order, and second-order reaction kinetics, respectively.

## 3. Results and Discussions

TiO_2_ is largely used as a photocatalyst for the degradation of organic materials, due to its high superior photocatalytic activity, low cost, and elevated chemical stability [33]. However, the use of titanium dioxide in the industrial process presents different disadvantages; the first is TiO_2_’s separation from the slurry after the water treatment. For this reason, a separation/recovery step is required. Another problem is represented by the tendency of the TiO_2_ particle to form agglomerates, with a consequent reduction of its surface area [34]. The combination of the membrane separation with the photocatalytic process represents an efficient, low-cost, eco-friendly technology, with great potential in wastewater treatment. In this work, high-flux inorganic photocatalytic membranes have been developed using the dip-coating method. The novelty of the work is represented by the study of the TiO_2_ suspension stability, considering the Turbiuscan Stability Index. The use of stable suspensions for the photocatalytic membrane preparation permits a reproducible method and, at the same time, a better distribution of the titanium dioxide nanoparticles in the membrane structure (due to a reduction of the agglomerate formation). This last aspect ensures an improvement of the photocatalytic activity, for better interactions between the active catalytic sites and the molecules of dye.

**Figure 3:**
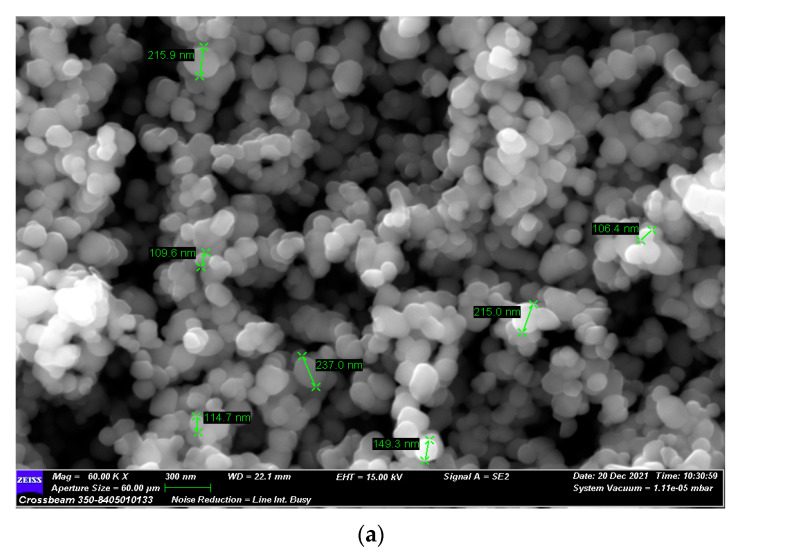

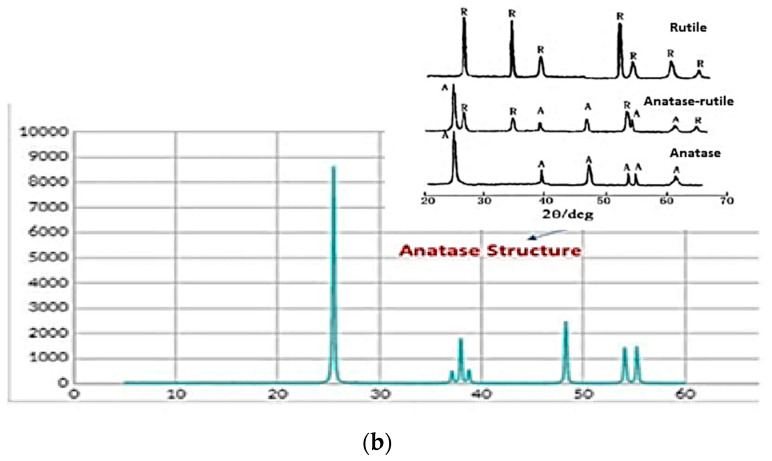
The structure analysis of commercial TiO_2_: (**a**) SEM picture and (**b**) XRD diffraction pattern.

Initially, the TiO_2_ powder was characterized by SEM analysis, and an average particle size of 150 nm was measured (see Figure 3a). TiO_2_ in nature exhibits different crystal structures: rutile, anatase, brookite, and srilankite [35]. When compared with the XRD reference patterns, the X-ray diffraction pattern of the TiO_2_ commercial powder was shown to be in the anatase state (typical peaks at 25° and 48°) [36] (see Figure 3b).

The titanium dioxide powder was used to prepare the suspensions for the dip-coating of the commercial tubular alumina membranes. The preparation of a stable suspension is very difficult, because the particles in the aqueous medium tend to aggregate, owing to van der Waals attractive forces. This determines the formation of clusters and their subsequent precipitation. There are several methods to suppress the agglomeration of the nanoparticles in a suspension, such as electrostatic or steric stabilization [37,38]. The electrostatic stabilization is obtained by changing the pH, and in this case, the van der Waals force attractions are counterbalanced by the repulsive Coulomb forces. The steric stabilization method involves using some additives (e.g., polymers) and their adsorption on the surface of the NPs prevents their agglomeration [39]. Considering these aspects, different suspensions were prepared (see Table 1), and the TSI trend over time for each of them was evaluated. The TSI values obtained for the suspensions prepared without additives are reported in Table 2.

**Figure 4:**
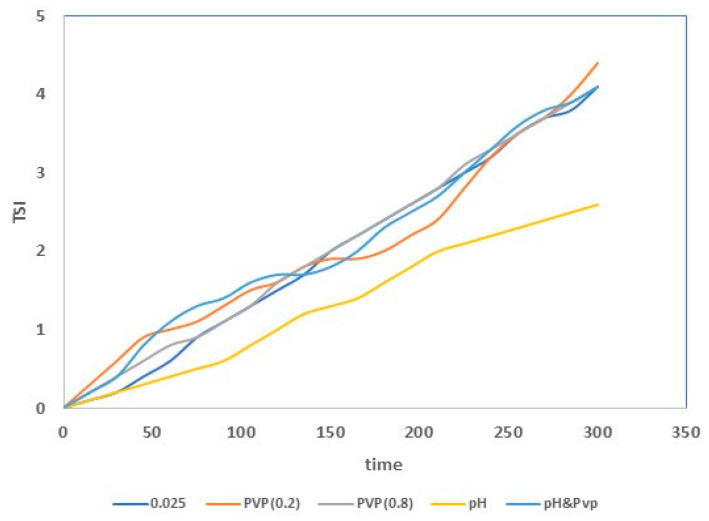
TSI values for the different prepared suspensions.

TSI values decreased with decreasing titanium dioxide concentrations, indicating higher stability, due to a reduction of TiO_2_ agglomerate formation (sedimentation and clarification phenomena suppression) [40,41]. Therefore, the suspension with a TiO_2_ concentration of 0.025 wt. % was the most stable. The stability of this suspension was further improved with the addition of nitric acid and PVP. The addition of the acid permitted an electrostatic stabilization, and the PVP addition permitted steric stabilization. The lowest TSI value was found for the suspension having a titanium dioxide concentration of 0.025 wt. %, and containing nitric acid (see Figure 4). The improved stability is related to the zeta potential (ZP) value of the suspension [42]. In particular, for ZP values close to zero (isoelectric point), the particles form agglomerates [43]. In contrast, for highly negative or positive values of ZP (more than +30 mV or less than −30 mV), the formation of agglomerates is avoided [43,44]. The zeta potential values for the suspensions (TiO_2_: 0.025 wt. %) prepared with and without additives are reported in Table 3. It is possible to observe that the acid’s presence determined a lowering of the zeta potential. In fact, the zeta potential value of this suspension was lower than −30 mV, and so the nanoparticles (negatively charged) repelled each other by electrostatic repulsion.

**Figure 5:**
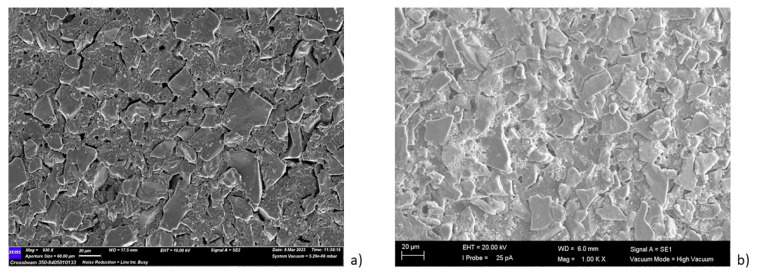

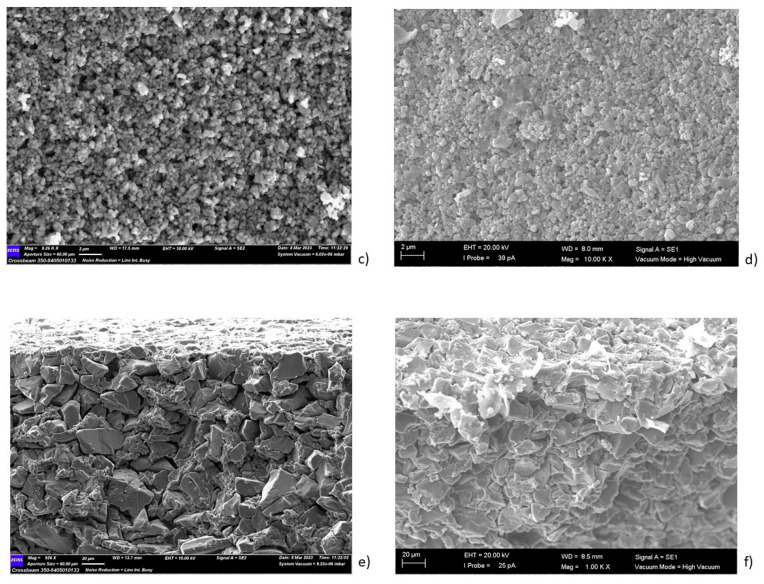
Morphological analysis of the (**a**) external surface, (**c**) internal layer and (**e**) cross-section of the pristine membrane; (**b**) the external surface, (**d**) internal layer and (**f**) cross-section of the photocatalytic membranes.

In addition, considering that the zero charge point of α-Al_2_O_3_ is 9.0 [45], the use of a suspension having a pH of four enabled the electrostatic interaction between alumina and titanium dioxide during the dip-coating. The SEM pictures of the pristine and photocatalytic membranes are reported in Figure 5.

**Figure 6:**
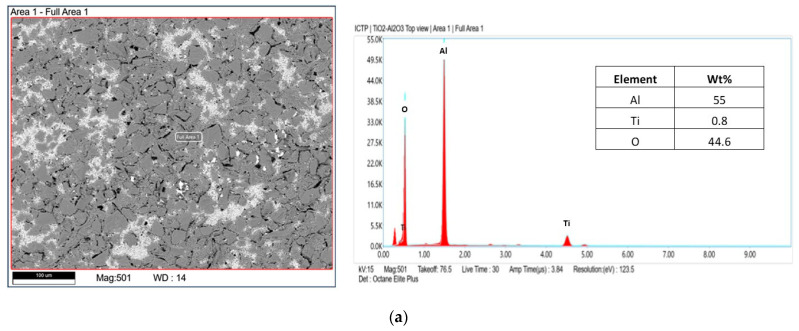

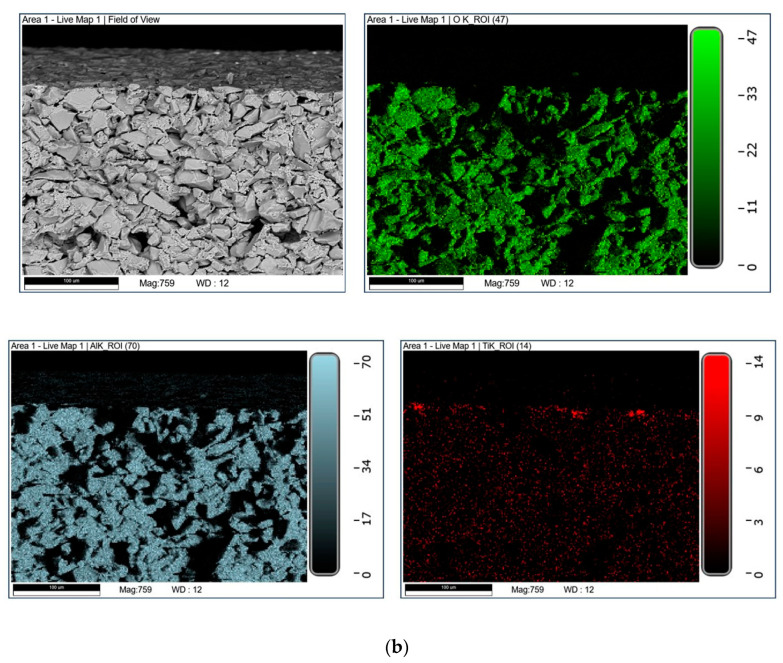
EDX analysis of the photocatalytic membrane; (**a**) top-view and (**b**) cross-section.

The tubular α-Al_2_O_3_ membranes exhibited an asymmetric structure. In particular, an external layer with an average pore size of 3 μm and a separate layer with a pore size of about 0.2 µm were detected. The SEM analyses of the photocatalytic membranes showed the presence of the TiO_2_ nanoparticles, which were mainly on the external surface. The EDX analyses confirmed this result. In fact, it was shown that the titanium dioxide was mainly present on the surface, and decreased along the cross-section (see Figure 6).

The hydraulic permeance of pristine membranes is about 1500 Lm^−2^ h^−1^ bar^−1,^ and after the dip coating, the membranes’ flux decreased by about 11%. The dip-coating method permitted the formation of the photocatalytic layer, mainly on the surface of the macroporous alumina membrane, with the possibility of avoiding a large reduction of the water permeability. A comparison with literature data, in terms of photocatalytic inorganic membranes prepared with the dip-coating method, is reported in Table 4.

A significant loss of permeability indicates the plugging of titanium dioxide into the pores of the alumina membranes [46,47,48] (coating of the surface is characterized by a small pore size). A smaller permeability decrease was observed when coating the membrane’s external surface (macroporus structure). In this case, it is possible to minimize the porosity reduction of the membrane (ensuring high fluxes), and, at the same, to preserve its separative layer.

**Figure 7:**
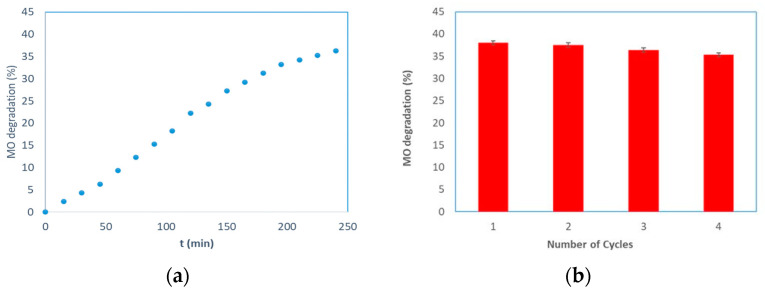

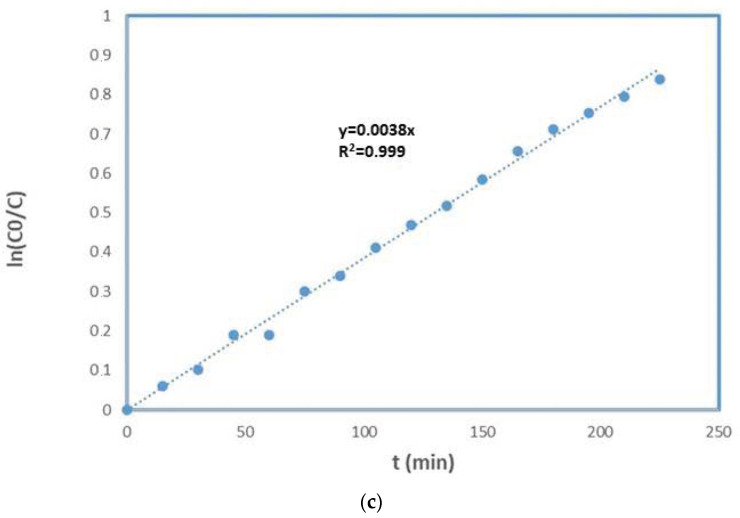
MO degradation by using the TiO_2_–Al_2_O_3_ membranes: (**a**) MO degradation versus time. (**b**) Reusability of the TiO_2_–Al_2_O_3_ membrane. (**b**) MO degradation after four successive reactions. Kinetics of methyl orange photodegradation: (**c**) ln(C_0_/C) versus time for the MO degradation. Operating conditions: C_0_ = 1 mgL^−1^; Volume of MO solution = 1 L; Stirring rate = 3 rpm; T = 25 °C; UV energy = 365 nm.

The photocatalytic activity of the prepared membranes was studied using the methyl orange a recalcitrant and carcinogenic azo dye. In Figure 7a, the MO degradation is shown as a function of time, obtained using a PMR operating in a continuous cross-flow mode. It is possible to observe that the degradation percentage increased by enhancing the UV exposure time. The dye degradation was 36% across the interval of time considered. The photocatalytic reaction was performed without UV irradiation for the first 30 min. In this case, a slight decrease in the MO concentration was detected due to the adsorption of MO on the surface of the alumina membrane. The adsorption between the MO and the alumina is favored, considering their zero charge points (MO = 2.5 [49] and α-Al_2_O_3_ = 9.0 [45]), and the pH (6.5) of the feed solution. The decrease of the MO concentration, achieved by turning on the UV light, indicates the positive effect of the generation of reactive species by the photocatalyst under UV irradiation. No photocatalytic degradation of MO was observed when a pristine membrane was used. The reusability of the photocatalytic membranes was tested by comparing the MO degradation during four successive reactions, each 4 h in duration; after each reaction, the plant was washed with distilled water. The obtained results are reported in Figure 7b. The experimental data showing the photocatalytic activity slightly decreased, which was probably due to the accumulation of organic materials (formed during the reaction) on the active sites of the TiO_2_ nanoparticles. Each repeated experiment exhibited a loss of efficiency of about 1%.

For evaluating the kinetic degradation of the MO, the experimental data were plotted versus time considering the Equations (7)–(9). A linear plot was observed by plotting the ln(C_0_/C_t_) versus time (R^2^~1), indicating that the degradation reaction of methyl orange followed the pseudo-first-order kinetic model [50,51] (see Figure 7c). In different papers, the same result was also found [52,53,54]. Finally, a comparison with literature data is reported in Table 5.

A comparison with literature data showed that the amount of TiO_2_ immobilized into the porous structure of the alumina tubular membrane influenced the performance of the process. Small or large amounts of photocatalytic particles negatively influenced the degradation process. This is explained, considering that a higher amount determines a reduction of both surface area and light absorption capacity, due to the formation of agglomerates [55,56]. In addition, if the amount of photocatalytic NPs is small, the numbers of active sites decrease, with a reduction of the MO degradation [57].

This work presents a key factor in the preparation of the photocatalytic membranes, by using the dip-coating to improve the stability of the suspension. In fact, the use of a stable suspension (in the interval time used for the membrane preparation) permits us to reduce the agglomerate formation and to achieve a better dispersion of the TiO_2_ NPs in the porous structure of the membranes ensure a better contact between dyes and catalytic sites.

## 4. Conclusions

In this work, the Turbiscan stability index was used for the first time to evaluate the stability of the suspensions used for preparing the photocatalytic membranes by the dip-coating method. The dip-coating, performed on the external surface of the alumina membranes, permitted the preparation of high-flux photocatalytic membranes. The photocatalytic performance of the membranes has been examined in a photocatalytic membrane reactor (PMR) under UV irradiation, and using methyl orange as a model recalcitrant dye. A degradation of 36% was detected after 4 h of reaction. The reusability of the photocatalytic membranes was also studied, and each repeated experiment exhibited a low-efficiency loss. Photodegradation of MO followed the pseudo-first-order kinetic model.

## Figures and Tables

**Table 1:** Composition of the titanium dioxide suspensions.

Sample	Inorganic Powder	Liquid	TiO_2_ Concentration (wt%)	Sonication	PVP Solution (V = 5 mL)	HNO_3_ (wt. %)	pH
1	TiO_2_	H_2_O	0.025	No	No	No	6.5
2	TiO_2_	H_2_O	0.25	No	No	No	6.5
	TiO_2_	H_2_O	0.10	No	No	No	6.5
4	TiO_2_	H_2_O	0.05	No	No	No	6.5
5	TiO_2_	H_2_O	0.025	Yes	No	No	6.5
6	TiO_2_	H_2_O	0.025	No	PVP(0.2 wt. %)	No	6.5
7	TiO_2_	H_2_O	0.025	No	PVP (0.8 wt %)	No	6.5
8	TiO_2_	H_2_O	0.025	No	No	Yes	4.5

**Table 2:** TSI for the TiO_2_ suspensions prepared without the additive.

TiO_2_ Suspension (wt. %)	TSI (3 h)	TSI (5 h)
0.025	2.4	4.1
0.05	2.6	4.5
0.10	4.3	6.7
0.25	4.4	7.0

**Table 3:** Zeta potential values for TiO_2_ suspensions with and without additives.

Sample	Zeta Potential [mV]
TiO_2_ suspension (0.025 wt. %; pH = 6.5)	−20.8
TiO_2_-PVP suspension (0.025 wt. %; pH = 6.5; PVP = 0.2 wt. %)	−24.4
TiO_2_-HNO_3_ suspension (0.025 wt. %; pH = 4.5)	−37.6

**Table 4:** Comparison with the water permeabilities of different inorganic photocatalytic membranes.

Sample	Before Coating Hydraulic Permeance (Lm^−2^ h^−1^ bar^1^)	After Coating Hydraulic Permeance (Lm^−2^ h^−1^ bar^1^)	Reduction (%)	Ref.
TiO_2_ film on α-Al_2_O_3_ membrane (pore size = 0.2 μm)	1800	150	92	[46]
N-doped TiO_2_ film on α-Al_2_O_3_ membrane (pore size = 0.2 μm)	3800	160	58	[47]
Si-doped TiO_2_ film on α-Al_2_O_3_ membrane (pore size = 0.1 μm)	1950	340	83	[48]
TiO_2_ on α-Al_2_O_3_membrane (asymmetric support: external pore size = 3 µm, internal pore size = 0. 2 µm)	1500	1330	11	This work

**Table 5:** Comparison with literature data.

Sample	MO (mg/L)	TiO_2_ (mg)	Irradiation Time (min)	MO Degradation (%)	Ref
PES-TiO_2_	10	1000	240	25	[55]
α-Al_2_O_3_-TiO_2_ *	6.5	54	60	20	[56]
γ-Al_2_O_3_-TiO_2_ *	2	20	-	50	[57]
PS-TiO_2_	1	0.3	180	4	[58]
α-Al_2_O_3_-TiO_2_ *	7.8	-	360	60	[59]
α-Al_2_O_3_-TiO_2_ **	1	10	240	36	This work

* TiO_2_, layer on the alumina membrane; ** TiO_2_, dispersion in alumina membrane.

## Data Availability

Data will be available on request.
